# Effectiveness of an Integrated Engagement Support System to Facilitate Patient Use of Digital Diabetes Prevention Programs: Protocol for a Randomized Controlled Trial

**DOI:** 10.2196/26750

**Published:** 2021-02-09

**Authors:** Katharine Lawrence, Danissa V Rodriguez, Dawn M Feldthouse, Donna Shelley, Jonathan L Yu, Hayley M Belli, Javier Gonzalez, Sumaiya Tasneem, Jerlisa Fontaine, Lisa L Groom, Son Luu, Yinxiang Wu, Kathleen M McTigue, Bonny Rockette-Wagner, Devin M Mann

**Affiliations:** 1 Healthcare Innovation Bridging Research, Informatics, and Design Lab Department of Population Health NYU Langone Health New York, NY United States; 2 Clinical Systems & Clinical Transformation Medical Center Information Technology Clinical Informatics Department NYU Langone Health New York, NY United States; 3 Department of Population Health NYU Langone Health New York, NY United States; 4 Division of Biostatistics Department of Population Health NYU Langone Health New York, NY United States; 5 NYU Rory Meyers College of Nursing New York, NY United States; 6 Division of General Internal Medicine Department of Medicine University of Pittsburgh Pittsburgh, PA United States; 7 Department of Epidemiology Graduate School of Public Health University of Pittsburgh Pittsburgh, PA United States

**Keywords:** mobile health, mHealth, eHealth, diabetes prevention, type 2 diabetes mellitus, mobile phone

## Abstract

**Background:**

Digital diabetes prevention programs (dDPPs) are effective behavior change tools to prevent disease progression in patients at risk for diabetes. At present, these programs are poorly integrated into existing health information technology infrastructure and clinical workflows, resulting in barriers to provider-level knowledge of, interaction with, and support of patients who use dDPPs. Tools that can facilitate patient-provider interaction around dDPPs may contribute to improved patient engagement and adherence to these programs and improved health outcomes.

**Objective:**

This study aims to use a rigorous, user-centered design (UCD) methodology to develop a theory-driven system that supports patient engagement with dDPPs and their primary care providers with their care.

**Methods:**

This study will be conducted in 3 phases. In phase 1, we will use systematic UCD, Agile software development, and qualitative research methods to identify *key user* (patients, providers, clinical staff, digital health technologists, and content experts) requirements, constraints, and prioritization of high-impact features to design, develop, and refine a viable intervention prototype for the engagement system. In phase 2, we will conduct a single-arm feasibility pilot of the engagement system among patients with prediabetes and their primary care providers. In phase 3, we will conduct a 2-arm randomized controlled trial using the engagement system. Primary outcomes will be weight, BMI, and A_1c_ at 6 and 12 months. Secondary outcomes will be patient engagement (use and activity) in the dDPP. The mediator variables (self-efficacy, digital health literacy, and patient-provider relationship) will be measured.

**Results:**

The project was initiated in 2018 and funded in September 2019. Enrollment and data collection for phase 1 began in September 2019 under an Institutional Review Board quality improvement waiver granted in July 2019. As of December 2020, 27 patients have been enrolled and first results are expected to be submitted for publication in early 2021. The study received Institutional Review Board approval for phases 2 and 3 in December 2020, and phase 2 enrollment is expected to begin in early 2021.

**Conclusions:**

Our findings will provide guidance for the design and development of technology to integrate dDPP platforms into existing clinical workflows. This will facilitate patient engagement in digital behavior change interventions and provider engagement in patients’ use of dDPPs. Integrated clinical tools that can facilitate patient-provider interaction around dDPPs may contribute to improved patient adherence to these programs and improved health outcomes by addressing barriers faced by both patients and providers. Further evaluation with pilot testing and a clinical trial will assess the effectiveness and implementation of these tools.

**Trial Registration:**

ClinicalTrials.gov NCT04049500; https://clinicaltrials.gov/ct2/show/NCT04049500

**International Registered Report Identifier (IRRID):**

DERR1-10.2196/26750

## Introduction

### Background

More than 80 million US adults are considered prediabetic. Without treatment, an estimated 15% to 30% will develop diabetes over the next 5 years [1]. The epidemic of prediabetes is driving the morbidity and mortality of the downstream manifestations of type 2 diabetes (DM2) and cardiovascular disease, of which there are 2 to 3 times increased odds in persons with prediabetes [2,3]. Thus, effective, and scalable management solutions are greatly needed.

Evidence-based interventions to prevent DM2 have focused on behavior change therapies including weight loss, dietary changes, and exercise. This focus on behavior change was in response to findings from seminal research in Finland and the United States, which demonstrated that intensive lifestyle changes (including a low-fat diet, 150 min per week of moderate exercise, and a target 7% weight reduction) were as effective as medication in preventing the progression to DM2 in at-risk patients [4]. In response to these findings, the Centers for Disease Control and Prevention (CDC) developed a national diabetes prevention program (DPP) in 2010 that was aimed at providing patients with comprehensive, research-based, cost-effective programs to help prevent diabetes. To date, more than 1500 organizations nationwide have partnered with the CDC to deliver DPP and more than 100,000 individuals have participated in one of these programs [5].

### Digital Diabetes Prevention Program Platforms

The DPP curriculum has been successfully adapted to a variety of digital platforms, known as digital DPPs (dDPPs). Several commercial dDPP vendors are currently available to consumers (Noom, Livongo, and Omada). To varying degrees, the core elements of these programs include (1) a structured lesson plan on diabetes prevention, weight loss, exercise, and other areas of lifestyle modification adapted from the CDC program; (2) a system of activity, steps, meal, or other feature tracking, which patients can either automatically or manually upload to their device; and (3) a personalized coaching and social support network.

Early data have demonstrated the effectiveness of dDPPs in achieving weight loss, A_1c_ reduction, and other key diabetes health outcomes at 6 months and 1 year [6-8]. These digital platforms also offer benefits to patients in terms of accessibility, convenience, and personalization, which make them attractive alternatives to the more resource-intensive in-person DPPs. This potential has been further highlighted by the COVID-19 pandemic, in which disruptions in continuity of care for chronic disease management and elevated barriers to in-person health activities such as fitness classes and group nutrition counseling have driven more people to digital platforms for health behavior change [9].

### Digital Health Engagement: Challenges for Patients and Providers

Despite the popularity and effectiveness of digital health interventions such as dDPPs, the integration of these tools into clinicians’ armamentaria for disease management and healthy behavior change has been limited [10]. The reasons for this include technical barriers, suboptimal user experience, entrenchment of practice habits and preferences, perceived administrative burden, and unfavorable reimbursement environments. In particular, user engagement in digital health tools represents a critical, but challenging, component of the effective translation of evidence-based behavioral interventions into pragmatic, scalable digital solutions [11,12]. Although regular interaction with digital health tools for weight loss and diabetes prevention has been shown to improve targeted health outcomes with a tendency toward a dose-response relationship [13-15], low rates of long-term engagement are known barriers to achieving and maintaining these outcomes [16-18]. Tools to identify and measure engagement have been lacking, in part, because of the lack of conceptual clarity and precision in defining the features of engagement, and gaps remain in the understanding of the links between specific engagement behavior and the achievement of target health goals [19-21]. For providers, poor integration of dDPPs into the existing health information technology (HIT) and electronic health record (EHR) workflows negatively impacts the ability to incorporate relevant aspects of the programs into patient care, communication, or education. This results in missed opportunities for comprehensive care delivery in diabetes prevention, as providers are unable to overcome barriers in technology, workflows, and competing priorities to effectively leverage digital health tools. Integrated clinical tools that can facilitate patient-provider interaction around dDPPs may contribute to improved patient engagement and adherence to these programs and improved health outcomes by addressing some of these issues.

### Objectives

Few studies have investigated integrating consumer-facing digital health programs such as dDPP platforms into existing clinical systems, such as the EHR, or into clinical workflows of ambulatory care practices to facilitate patient-provider interaction with these tools. We hypothesize that existing digital technology, such as text messaging systems, patient portals, and EHR integrations, can support both clinicians and patients by improving communication, education, and shared decision making around digital behavior change tools. The purpose of this study is to design and test a novel clinical tool to enhance engagement with digital behavior change efforts in diabetes prevention.

## Methods

### The Integrated Framework for the Development of Digital Health Behavior Change Interventions

**Figure 1:**
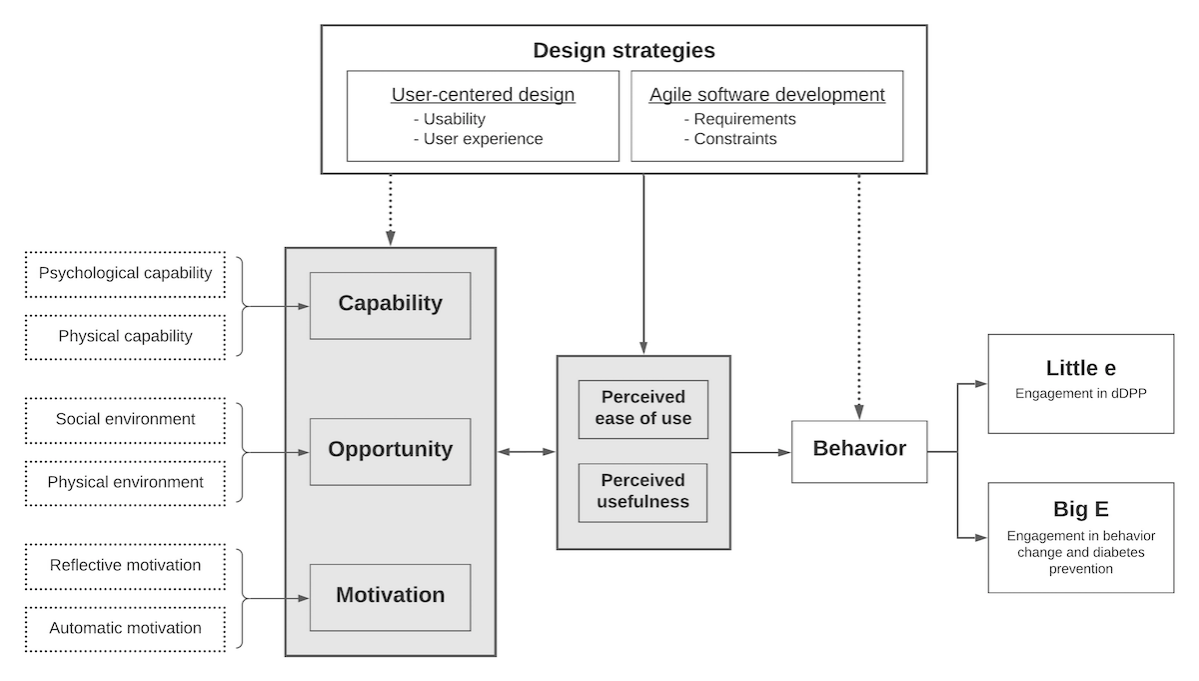
The integrated framework for the development of digital health behavior change interventions. dDPP: digital diabetes prevention program.

The dDPP engagement intervention uses an integrated framework that combines established theoretical models for behavior change with effective digital health implementation strategies (Figure 1). This combination leverages both theoretical and pragmatic approaches to the development, implementation, and evaluation of digital behavior change tools and provides a structure for both outcomes and process measures.

This integrated framework adapts the Capability, Opportunity, and Motivation Model of Behavior Change (COM-B); the Technology Acceptance Model (TAM); and the Johnson and Johnson (J&J) approach to engagement in digital behavior change interventions. COM-B is a comprehensive model developed by Michie et al [22] who identify 3 components of behavior (capability, opportunity, and motivation) that may be activated and/or suppressed to elicit targeted actions and effect change. This model is widely used in behavior change health research and has been proven effective for designing health interventions targeting disease prevention [23-26]. The TAM is an information technology framework based on the Theory of Reasoned Action that conceives of beliefs and attitudes as determining intentions, which in turn dictate behavior. It asserts that perceptions of usefulness and ease of use by end users will directly influence intention to use new technology, leading in turn to its adoption [27,28]. TAM and its subsequent versions TAM2 and TAM3 have been widely applied to explain the adoption of HIT [29,30]. The J&J approach to digital health engagement derives from the belief that user engagement with digital behavior change interventions (DBCIs) is a precursor to improved health outcomes. Engagement with DBCI can be divided into 2 types—*Big E* and *Little e*—with *Big E* describing engagement with targeted health behavior (eg, weight loss) and *Little e* representing engagement with the digital behavior change intervention itself (eg, weight tracking) [19]. This combined framework leverages the core relevance of older, well-accepted models (particularly TAM, which was first developed in the 1980s) while acknowledging the innovative contributions of later theories that address new areas of exploration in digital behavior change, particularly digital app development.

**Figure 2:**
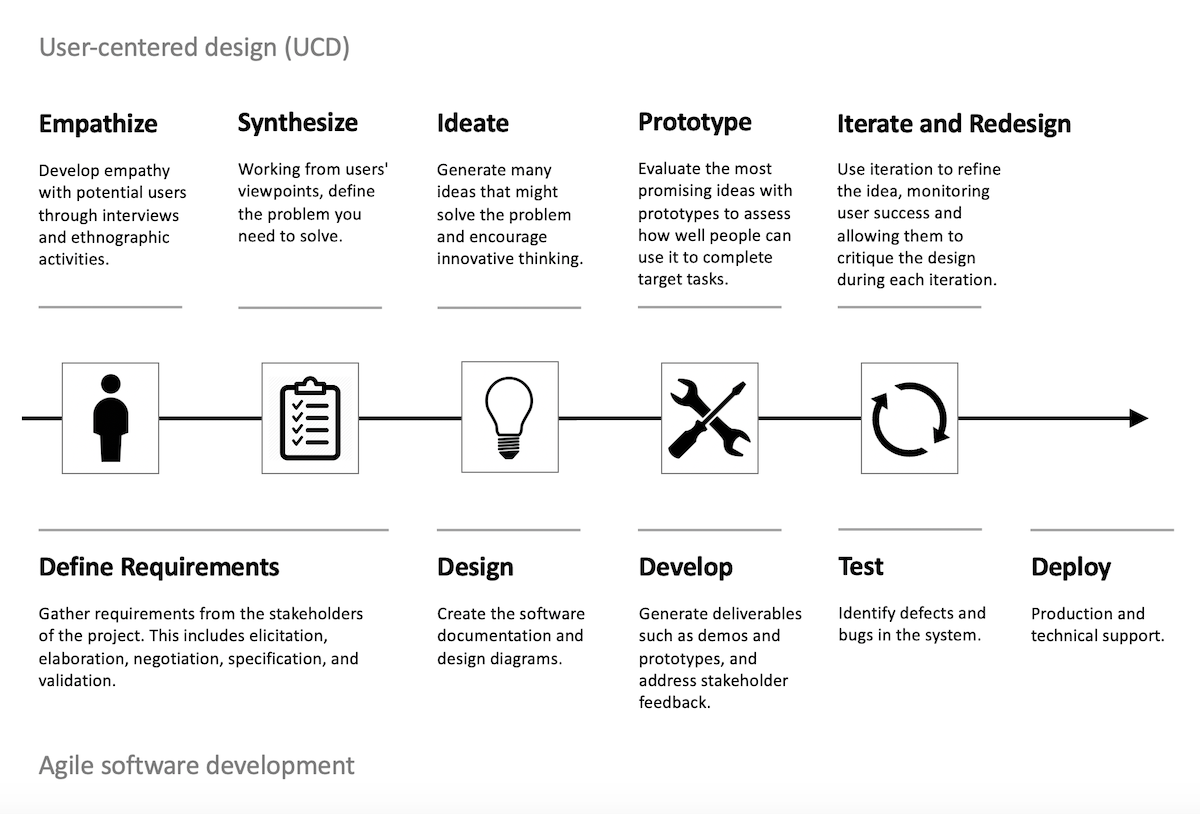
User-centered design and Agile software development.

In addition to our theoretical model, we use complementary strategies of user-centered design (UCD) and Agile project management for the design, development, and implementation of our engagement intervention (Figure 2). UCD and the related Design Thinking (DT) process have emerged as novel frameworks for product development and research in health care, particularly in the areas of health care technology and digital health product development [31]. UCD uses repeating cycles of ideation, prototyping, testing, and refinement to develop digital health interventions in collaboration with end users (eg, patients, providers, health administrators, technologists, other stakeholders), with the goal of building products that are appropriate, acceptable, and usable for those users. The iterative nature of UCD also allows for continual innovation, adaption, and refinement of products over time. Agile is a process derived from software development that involves the identification and review of key requirements (eg, needs, preferences, expectations of users) and the continuous generation of partial deliverables for stakeholders and end users. Agile processes include users at every stage of product development and allow stakeholders to be actively involved in the development process, from inception to implementation. Decisions and changes to a product are discussed among the multidisciplinary team to arrive at the best solution for the study intervention. As the intervention evolves, the computational system will evolve to address the changes. Applying the strategies of UCD and Agile facilitates the development of targeted, acceptable, and adaptable digital health interventions, thereby improving the likelihood of both effectiveness and adoption and adherence by users.

### dDPP Platform: Noom

Our study (NCT04049500) will use the commercially available application Noom to provide patients with a dDPP platform. Noom is a mobile health behavior change lifestyle app based on the CDC’s DPP that allows users to log their meals, weight, and exercise and physical activity minutes. Noom connects users with an individual behavior change coach and support group and provides a DPP curriculum through daily articles that cover topics including diet, exercise, and healthy behavior psychology. The core philosophy of Noom is to work with the user to adopt a healthier lifestyle in a way that best fits the user’s individual life. Preliminary studies of Noom showed high levels of effectiveness and acceptability compared with in-person DPPs. In overweight or obese adults with prediabetes, participation in Noom was associated with significant weight loss at 24 and 65 weeks, which exceeded the CDC DPP 5% weight loss requirement [7,32]. In one study, participants exhibited a dose-dependent response with greater mean weight loss at 65 weeks in those who engaged more in the program [32]. In addition, certain metrics of in-app engagement such as weekly logged meals, weigh-ins, and group posts were shown to predict weight loss [32]. Noom is a well-established commercial dDPP platform with a growing market share and considerable brand recognition and robust customer and technical support.

### Study Overview

This study will consist of 3 phases.

- *Phase 1* will combine a theory-based approach to behavior change with UCD and Agile software/technical strategies to
  Identify the needs, requirements, preferences, and constraints of users (patients, clinical providers, ambulatory practice management and technologists, dDPP coaches, and technical teams).
  Co-design a patient- and provider-facing engagement intervention (ie, mobile app, text messaging system, EHR visualization) to support patients’ use of the dDPP and assess its usability via iterative prototyping and user testing.
- *Phase 2* will consist of a single-arm feasibility pilot among patients with prediabetes using the engagement intervention tool and a validated third-party vendor dDPP platform to assess the preliminary effectiveness of the engagement system and further refine the intervention.
- *Phase 3* will consist of a two-arm randomized controlled trial (RCT) to evaluate the impact of the engagement intervention tool plus the dDPP platform versus the dDPP platform alone on health outcomes (weight, BMI, and A_1c_) and engagement in the dDPP.

### Inclusion and Exclusion Criteria for Patients and Providers

Eligible patients will have been diagnosed with prediabetes (A_1c_ level of 5.7-6.4 in the last year) or have risk factors for diabetes (obesity and family history). In addition, they must have access to smartphones or internet-connected tablets and be able to read and write English. We will exclude patients who have ever been diagnosed with diabetes (A_1c_ level>6.4) or those with contraindications to weight loss, dietary adjustments, or moderate physical activity. Patients whose weight may vary considerably over the study’s timeframe for reasons other than the intervention (eg, cancer, pregnancy, ascites, severe congestive heart failure) and patients with severe psychiatric disease, dementia, or vision or other impairments that would prevent them from being able to access and use the dDPP app will also be excluded.

Eligible providers will have at least 2 years of experience providing care to patients with prediabetes or risk factors for diabetes in the outpatient setting.

### Phase 1: Intervention Design

#### Study Design

In this phase of the study, we will employ UCD techniques to identify use cases, needs (requirements), preferences, and constraints of a targeted digital engagement intervention and use Agile methodologies to guide the intervention’s technical development.

#### Setting and Participants

This study will be conducted within the ambulatory practice network of New York University Langone Health (NYULH). The network cares for more than 7.64 million diverse patients throughout New York, New Jersey, and Florida and includes more than 8000 health care providers. Practice sites include academic faculty practices, community clinics, and Federally Qualified Health Centers. The entire ambulatory network shares a single integrated EHR (Epic).

Enrollment for the intervention design and development phase will include 25 to 30 diverse stakeholders or *key users*—patients and health care providers who meet our inclusion criteria, health technologists, behavioral change theorists, and dDPP vendor staff (coaches and developers). Sample size estimates for this phase were based on best practices for maximizing the power of qualitative research, which recommend 6 to 8 participants per qualitative method, with additional participants as needed to achieve goal data collection and/or thematic saturation. Previous UCD studies suggest that 2 to 3 cycles of user testing are required to reach saturation [33].

#### Procedures and Data Collection

In this phase, we will apply the UCD model of *empathize, define, ideate, prototype, and test* to iteratively gather information, define, design, and refine the engagement intervention. This method will be applied over several cycles until a minimum viable product (MVP) of a workable intervention prototype is developed. The tools deployed for this process (eg, DT workshops, think-alouds, and usability testing) have been described extensively elsewhere and have been used with success by this and other research teams in the development of digital health technologies [33-37].

In the *empathize* and *define* stage, we will conduct focus groups and interviews to capture experiences and baseline needs of key users including patients, providers, technologists, and content experts in diabetes prevention, digital health engagement, and behavior change theory. This information will be used to inform the focus of the subsequent stages. In the *ideate* stage, we will use a series of structured DT workshops developed in response to results from the previous stage to engage a multidisciplinary group in the organized predesign of possible intervention solutions. Specific ideation sessions will focus on the patient- and provider-facing components of the engagement intervention and interactions of the intervention with the commercial dDPP platform.

In the *prototype* and *test* stages, a select number of solutions will be chosen by the multidisciplinary group and the research team for further development to be undertaken by the research and technical teams. These prototypes will undergo a series of structured think-aloud and usability testing sessions with key users (patients and providers) and will be iteratively refined based on results from these sessions until no further substantive changes are required and an MVP is developed. Usability testing will include both the intervention itself and the intervention integrated with the commercial dDPP platform. Usability testing participants will be asked to complete demographic surveys and pre- and posttesting surveys.

#### Data Analysis

Qualitative data from interviews, focus groups, DT workshops, and usability testing will be recorded, transcribed, and coded both deductively to evaluate relevant domains of our integrated theoretical framework (eg, user requirements, preferences, and constraints; barriers and facilitators to tool use; ease of use, usability) and inductively to identify emergent themes and concepts. Coders will meet to review their coding, conduct team debriefing meetings, and reach a consensus on code names and meanings. Once all transcripts have been collaboratively coded, analytic domains will be identified and major and minor thematic areas will be described. Quantitative data from user testing surveys will be analyzed using basic statistical methods to identify significant associations between intervention use and relevant demographics.

Results from the qualitative and quantitative data analysis will be used to identify key *user stories*, a core technique in Agile methodologies for the identification of units of technical development work through the lens of a user [38,39]. User stories will be converted by the technical team into a series of discrete technical requirements and constraints that will be used to inform the technical build of the engagement intervention. User stories, requirements, and technical work or *tasks* will be tracked and completed using Agile project management software (ClickUp).

### Phase 2: Feasibility Pilot

#### Study Design

Following phase 1 development of an intervention MVP, we will conduct a single-arm pilot test to further evaluate the feasibility and the process of implementing the intervention. The findings will inform additional refinements in advance of the RCT.

#### Setting and Participants

The study will take place across 2 ambulatory care practices within the NYULH ambulatory network, selected based on their practice size and volume of patients with prediabetes. In total, 20 patients who meet the inclusion criteria will be enrolled; 5 to 8 providers whose patients were enrolled in the study will also be enrolled. The sample size is based on best practices for maximizing the power of qualitative research and estimates of the number of users needed to inform additional rounds of prototype refinement. For feasibility studies, 24 to 50 participants are generally recommended [40]. Additional patients and providers may be recruited until thematic saturation is met and/or no further refinements to the intervention are identified.

#### Procedures and Data Collection

Eligible patients will be identified through (1) the review of patient data in the EHR and (2) provider referrals. Eligible participants will be consented by a member of the research team. Consented patients will be enrolled in the commercial dDPP and receive the patient-facing engagement intervention developed in phase 1. Patients will receive information and training on both the national DPP and the intervention dDPP and will be guided through downloading and enrolling in the dDPP platform. Patients will also receive wireless connected step trackers and weight scales and will be instructed on how to connect their devices to their dDPP account and upload their health data. Eligible physicians whose patients have been enrolled in the study will be included in the study and will receive the provider-facing engagement intervention developed in phase 1.

Patients and providers will be asked to complete surveys at various points throughout the study.

Patient-level data include the following:

- Participant demographics: a self-report survey will collect patient sociodemographic data including age, sex or gender, race or ethnicity, and occupation. This will be compared with the patient data available in the EHR.
- Baseline *engagement readiness* survey: a self-report survey of areas related to digital engagement and health behavior change, including digital literacy, technology readiness, disease self-management and self-efficacy, quality of life, time management, and perceptions of their provider.
- Use behavior: patients’ dDPP use behavior will be measured by the research team at regular intervals throughout the study period, including the following dDPP features: meals logged, steps logged, exercise and/or physical activity logged, weights logged, and interactions with other dDPP features. Patients will also complete quarterly self-reports of engagement in the dDPP, including features used and motivation to use features.
- TAM survey: self-reported responses to questions derived from the validated TAM survey that assesses the perceived ease of use, usefulness, and quality of the patient-facing engagement intervention. This survey will be administered quarterly throughout the patients’ participation in the study.
- COM-B survey: a self-report of questions adapted from the well-established COM-B framework and related questionnaires, assessing capability, opportunity, and motivation of patients to (1) use the dDPP and (2) engage in health behavior change around diabetes prevention.

Provider-level data include the following:

- Provider demographics: a self-report survey will collect relevant provider demographics, including years of practice, practice type, and patient panel information.
- Use behavior and usability testing: providers will be interviewed at regular intervals regarding their use of the provider-facing engagement intervention, impact on patient management, impact on clinical workflows, and overall experience and evaluation.
- TAM survey: a self-report of questions derived from the validated TAM survey that assesses the perceived ease of use, usefulness, and quality of the provider-facing engagement intervention. This survey will be administered quarterly throughout the providers’ participation in the study.

#### Data Analysis

We will use descriptive statistics to summarize all patient- and provider-level outcomes and assess their relationship to patient engagement and activity data derived from the dDPP platform. All qualitative data collected via surveys will be analyzed using deductive and inductive (grounded theory) approaches, as described in phase 1. The results of quantitative and qualitative analyses in phase 2 will inform intervention approaches and assessments in phase 3.

### Phase 3: RCT

#### Study Design

In phase 3, we will conduct a two-arm pragmatic RCT to evaluate the impact of the engagement intervention tool plus the dDPP platform versus the dDPP platform alone on relevant prediabetes health outcomes and patient engagement in the dDPP.

#### Setting and Participants

The study trial will use a practice-level cluster-randomized design, with 1:1 randomization of 40 primary care practices resulting in 20 clinics per study arm. Randomization will be stratified by clinic size to ensure even distribution of different-sized clinics to the two study arms. The proposed sample size provides 80% power to detect a 30% increase in dDPP session completion and a 2 kg increase in 12-month weight loss in the intervention arm relative to the control. This calculation assumes 20% attrition, two-sided tests with a type I error rate of 0.05, and an intraclass correlation coefficient of 0.05.

We will recruit patients who meet the inclusion criteria with primary care providers at the study sites through the patient portal, an increasingly common and effective approach for recruitment [41-43], and through population-based outreach from NYULH prediabetes registries with assent from primary care providers. Intervention participation will last 12 months.

Patients enrolled in the intervention arm will receive access to the dDPP platform and the engagement system developed and refined in phases 1 and 2. Patients in the control arm will receive the dDPP alone. Providers in both arms will receive information on the study and educational material for their patients. Patients will receive enrollment and onboarding information and training for dDPP, the engagement system, and the home devices (weight scales and pedometers or fitness trackers), as outlined and refined in phase 2. In addition to the home devices, upon enrollment, patients will be provided a home A_1c_ testing kit and instructions for its use for the study.

#### Procedures and Data Collection

Eligible patients will be identified and enrolled as in phase 2, with applicable modifications as identified through phase 2 study design review and optimization.

The main health outcomes assessed in phase 3 are listed in Table 1 and correlate to health measures most commonly monitored in patients with prediabetes and goal outcome measures assessed by the dDPP.

**Table 1:** Phase 3 study outcomes.

Construct and measure				Data source		Collected at (months)
**Clinical outcomes**						
	Weight reduction (kg)		Noom (via a wireless scale)		Baseline, 6, and 12	
	Physical activity (steps per day)		Noom (via a wireless pedometer)		Baseline, 6, and 12	
	A_1c_ (%)		A_1c_ home test kit		Baseline, 6, and 12	
**Engagement outcomes**						
	**Patient engagement**		Noom		Weekly	
		Number of dDPP^a^ log-ins	Noom		Weekly	
		Number of dDPP lessons completed	Noom		Weekly	
	Perceived provider involvement with dDPP progress		Patient portal 1-item survey		3, 6, 9, and 12	

^a^dDPP: digital diabetes prevention program.

Changes in body weight will be our primary weight-based outcome. Second, we will assess the achievement of a 7% weight loss goal (the DPP weight goal). Weight will be collected from a Bluetooth-linked wireless weight scale, a validated process that automatically reports weigh-ins to the dDPP platform server [6,44-46]. In addition to providing the scale and weight measurement protocol, regular checks of weight data by research staff will be conducted to identify and follow up on values that appear invalid and comparisons with EHR-based data from clinical visits during the study period. Physical activity will be assessed using validated accelerometers or pedometers integrated into the dDPP platform [47-50]. A_1c_ will be assessed using home A_1c_ devices, which have been shown to be safe and equivalent to laboratory testing and are increasingly used in pragmatic digital studies to avoid unnecessary burden on participants [44,51,52].

Patient engagement in the dDPP will be measured using data on log-ins, lesson completion, feature interactions, and messages with coaches and social groups. These data will be reported at regular intervals from the dDPP using a secure application programming interface. Perceptions of engagement will be assessed at both patient and provider levels via a survey at 6- and 12-month intervals. To assess the determinants, process, and outcome measures associated with our theoretical model (capability, opportunity, motivation, ease of use, and usability) and relevant implementation outcomes (acceptability, adoption, cost, and sustainability), patients and providers will be asked to complete the surveys outlined in phase 2, with applicable modifications as identified through phase 2 design review and optimization. Data collection will occur at study enrollment (baseline) and 6- and 12-month intervals.

#### Data Analysis

All data will be descriptively summarized using frequencies and percentages for categorical variables and means and SDs for normally distributed data or median and IQR for skewed continuous variables. All available data will be included in data listings and tabulations with the number of missing values indicated. All analyses will follow the principle of intention-to-treat. Before analyzing the data, we will compare drop-out and missing data across study arms to assess whether any patient characteristics were associated with the missing data and, if necessary, perform additional analyses using multiple imputation methods.

The primary clinical outcomes (weight reduction or BMI, physical activity, and A_1c_) will first be analyzed as continuous variables using generalized estimating equation (GEE) models. Each GEE model will include a categorical indicator variable for the randomized study arm (control as reference), a variable corresponding to measurement time (baseline as reference), and an interaction term of the 2 variables. We will explore adjusting for any baseline demographics, use behavior, and survey responses that may be unbalanced between study arms. A Bonferroni-Holm correction method for multiple comparisons will be used to control the type I error rate of 0.05. A similar GEE model with a logit link function will be used to analyze the achievement of a 7% weight loss as a binary dependent variable.

We will also explore the extent to which engagement with the program acts as a mediator. We propose using a single mediator causal model approach with bootstrap-derived confidence intervals to measure the intervention’s effect on weight loss, physical activity, and A_1c_ through the specific mechanism of patient engagement.

#### Privacy and Security

Privacy and security of users’ data will be maintained by both the primary research team and the dDPP vendor in accordance with institutional and industry standard practices. We will ensure that participating patients and providers are informed of potential data requirements, usage, storage, and safety policies related to their study participation and follow standard procedures to address privacy breaches if they occur. All trial data will be saved on a dedicated server, available only to the study staff. Data will not be shared with third parties.

#### Informed Consent and Ethics Approval

Approval will be obtained through the NYULH Institutional Review Board. Participation in this trial is voluntary, and all eligible patients will be informed about the aims, risks, and benefits of the trial. Patients will be provided with written information and a consent form and given time to review the materials fully and ask questions before consenting. All patients can decline to participate in this trial and can withdraw consent at any time without penalty.

## Results

The project was initiated in 2018 and funded in September 2019. Enrollment and data collection for phase 1 began in September 2019 under an Institutional Review Board (IRB) quality improvement waiver granted in July 2019. As of December 2020, 27 patients have been enrolled and first results are expected to be submitted for publication in early 2021. The study received IRB approval for phases 2 and 3 in December 2020, and phase 2 enrollment is expected to begin in early 2021.

## Discussion

This proposed research will identify the needs and perspectives of key stakeholders in diabetes prevention management and incorporate them into the design and development of a targeted solution to support patient engagement and clinical integration of dDPP platforms. We will use the findings of this study to inform a larger scale study to assess the effectiveness and implementation of this intervention in routine ambulatory practices and diabetes prevention care. This type of technologically integrated support system has the potential to improve both patient engagement in an evidence-based dDPP and the experience of care by providing more seamless access to crucial elements of diabetes prevention for both patients and providers. This in turn may facilitate both disease management and the patient-provider relationship and ultimately improve health outcomes.

### Strengths

The strengths of this research include its pragmatic, multiphased UCD, which allows for iterative development and testing of digital interventions before deployment in a full RCT, thereby improving the likelihood of successful deployment and reducing the risks of technical problems, bias, or error in the clinical trial phase. Our use of an integrated theoretical framework that combines behavioral theory with UCD and Agile approaches will ensure that our intervention is both pragmatically and theoretically appropriate. Our partnership with a well-established third-party dDPP vendor leverages existing technology that has considerable market penetration and brand recognition and has been effectively user tested and validated in the consumer marketplace, rather than requiring the development of an in-house dDPP product. The extensive experience of this research team in developing, deploying, and evaluating digital health technologies will facilitate optimal study rollout [36,37,53-61].

### Limitations

There are several potential limitations to this study. First, we are only recruiting a small number of patients in phase 1 to identify the requirements of our intervention, which may not be representative of the broader population of patients with prediabetes in our health system. Therefore, it is possible that the features of our intervention will not apply to a larger or more diverse population. To address this, we have partnered with our system’s patient advisory committee, which is composed of representative samples of patients who act as patient advocates in research study design. The planned randomized trial will help us gain additional insights into the generalizability of our intervention. Second, although we are able to adapt the components of our intervention to the requirements of our users, we are unable to make changes to the third-party dDPP platform we have partnered with. It is possible that barriers to engagement and health behavior change will be driven by features of the dDPP platform, rather than our intervention. To address this, our process and outcome instruments will explicitly include questions of usability, ease of use, user experience, capability, opportunity, and motivation for both the *Little e* interventions (our engagement system *and* Noom) and *Big E* (diabetes prevention and lifestyle modification) to allow for analysis of each element. Finally, although the science of patient engagement is becoming more robust, there is still incomplete understanding of the moderators and mediators of engagement in digital applications, particularly digital health tools. It is possible that our study measures and instruments will not completely capture the nuances of user experiences, preferences, drivers, and behavior patterns that comprise engagement in digital behavior change technology or that then translate to behavior change itself. We have attempted to address this by enlisting experts in diverse fields of behavior change, patient engagement, behavioral economics, and digital technology and elsewhere to develop theoretically grounded, contextually relevant methods and measures.

### Conclusions

Our findings will help develop, evaluate, and validate technology that facilitates patient engagement in digital behavior change interventions for diabetes prevention and integrates dDPP platforms into existing clinical workflows for providers. Integrated clinical tools that can facilitate patient-provider interaction around dDPPs may contribute to improved patient adherence to these programs and improved health outcomes by addressing barriers faced by both patients and providers. Further evaluation with pilot testing and a clinical trial will assess the effectiveness and implementation of these tools.

## Abbreviations

CDC: Centers for Disease Control and Prevention
COM-B: Capability, Opportunity, and Motivation Model of Behavior Change
DBCI: digital behavior change intervention
dDPP: digital diabetes prevention program
DM2: type 2 diabetes mellitus
DPP: diabetes prevention program
DT: Design Thinking
EHR: electronic health record
GEE: generalized estimating equation
HIT: health information technology
IRB: Institutional Review Board
J&J: Johnson and Johnson
MVP: minimum viable product
NYULH: New York University Langone Health
RCT: randomized controlled trial
TAM: technology acceptance model
UCD: user-centered design
